# EP20 Hypercalcaemia in sarcoidosis

**DOI:** 10.1093/rap/rkaa052.019

**Published:** 2020-11-03

**Authors:** Tanya Chopra, Gordon MacDonald

**Affiliations:** Royal Berkshire Hospital, Reading, United Kingdom

## Abstract

**Case report - Introduction:**

Sarcoidosis often classically presents as Lofgren’s syndrome in up to 30% of cases, a triad of erythema nodosum, bilateral hilar lymphadenopathy and polyarthritis. However, the lack of identification and awareness of extrapulmonary manifestations of sarcoidosis can often lead to delayed diagnosis and treatment.

In sarcoidosis, hypercalcaemia is a feature in only 10-20% of all cases. However, the manifestation of hypercalcaemia may be the first presentation of sarcoidosis in patients who do not show the classical features of acute sarcoidosis.

**Case report - Case description:**

A 38-year-old man presented with a 5-month history of profound fatigue, poor concentration, and non-specific joint pains. He reported earlier swelling of his ankles and feet. He had lost 1 stone in weight over the last month. There was no history of fever or night sweats. He smoked 10 cigarettes per day but was otherwise fit and well.

On examination urine dipstick testing was negative. There was no evidence of lymphadenopathy. Cardio-respiratory and abdominal examinations were unremarkable. Examination of his skin and joints was also unremarkable. There was mild non-tender ankle oedema.

His first blood tests showed a raised adjusted calcium of 3.25 and a raised white cell count of 11.8, with an eosinophilia of 0.75. Other preliminary blood results were unremarkable (normal Hb, U+Es, LFTs, CRP, ESR, RF, anti-CCP, ANA and TFTS). His chest X-ray was reported as clear.

His PTH was appropriately suppressed and vitamin D level was adequate with normal urinary calcium and normal serum protein electrophoresis. Serum ACE level was raised at 114 (normal 8-52). PTH related peptide test was not available.

A CT chest abdomen and pelvis scan carried out to rule out malignancy was normal with no notable lymphadenopathy. A subsequent PET CT scan was normal.

Acutely, his hypercalcaemia was treated with IV fluids and IV pamidronate. Although his calcium rapidly normalised, he reported feeling only 10% better. He complained of ongoing ankle pain. An MRI scan of both ankles with contrast showed mild synovitis of ankle, subtalar and talonavicular joints. There was also evidence of tenosynovitis.

Given the constellation of hypercalcaemia, raised serum ACE level and ankle synovitis on MRI scan, he was treated for sarcoidosis with prednisolone 20mg. This led to a rapid improvement in his symptoms and normalisation of serum ACE. He was started on azathioprine as a steroid-sparing agent.

**Case report - Discussion:**

In cases series, hypercalcaemia due to sarcoidosis accounts for only 6% of all hypercalcaemic patients. The mechanism of hypercalcaemia in sarcoidosis is thought to be via activated pulmonary macrophages and sarcoid lymph node granulomas which upregulate the enzyme 1-alpha hydroxylase, resulting in the increased formation of calcitriol (1,25(OH)_2_D_3_). This increases calcium absorption from the gastrointestinal tract, stimulates renal calcium reabsorption and promotes calcium release from skeletal stores, causing hypercalcaemia.

This case was particularly unusual as earlier literature suggests that sarcoidosis-associated hypercalcaemia is a result of activated pulmonary macrophages and sarcoid granulomas. However, this patient had significant hypercalcaemia without any radiological lung involvement or granulomata, posing the question whether there are other pathways causing hypercalcaemia in sarcoidosis.

Hypercalcaemia without pulmonary involvement may be due to the presence of small amounts of sarcoid granulomata in extra-pulmonary locations such as the porta hepatis. These may not be as easily detectable on radiological investigations but may contribute to the upregulation of 1-alpha hydroxylase and subsequent hypercalcaemia. Another explanation for the significant hypercalcaemia in this patient may be due to the production of parathyroid hormone-related peptide (PTHrP) from sarcoid granulomas and bone marrow, which upregulates renal 1-alpha hydroxylase enzymes and increases the formation of calcitriol.

There was no area to obtain a tissue biopsy given the normal CT and PET CT scans, resulting in a greater reliance on history, examination, and serological investigations.

In addition, 30-50% of all patients with sarcoidosis have hypercalciuria, yet this patient interestingly had only an isolated hypercalcaemia with a normal urinary calcium.

**Case report - Key learning points**
 

1. Hypercalcaemia is rare in the absence of pulmonary involvement with only 10 cases reported in literature.
2. Although non-specific, an elevated serum ACE level may be a useful pointer to the diagnosis of sarcoidosis in the absence of other classical signs.
3. In this case, granulomatous tissue responsible to produce 1,25(OH)_2_D3 might be below the limits of radiological detection. Production may originate from extra-pulmonary sarcoid granulomatous tissue such as in the porta hepatis.
4. Another possible mechanism for hypercalcaemia may be the production of PTHrP which has been reported in sarcoid tissue specimens and in the bone marrow.

